# Mobile Mental Health Apps in China: Systematic App Store Search

**DOI:** 10.2196/14915

**Published:** 2020-07-27

**Authors:** Huifang Yin, Klaas J Wardenaar, Yuhao Wang, Nan Wang, Wenjin Chen, Yan Zhang, Guangming Xu, Robert A Schoevers

**Affiliations:** 1 Tianjin Anding Hospital Tianjin China; 2 Department of Psychiatry, Interdisciplinary Center Psychopathology and Emotion regulation University Medical Center Groningen University of Groningen Groningen Netherlands

**Keywords:** smartphone, app, mobile mental health, mental illness, mental health problem, China

## Abstract

**Background:**

Smartphones have become ubiquitous in China, offering a promising way to deliver mental health interventions; however, little is known about the current use and characteristics of smartphone apps for mental health.

**Objective:**

The purpose of this study was to gain insight into mobile mental health apps available in China as of December 2018.

**Methods:**

A systematic search was conducted to identify and evaluate the most downloaded apps from iOS and Android platforms. Apps were categorized according to their main purpose and downloaded to evaluate their content. Each app’s affiliation, cost, target users, information security, and evidence-based nature were evaluated.

**Results:**

Of the 172 unique apps that were identified, there were 37 apps (21.5%) for psychological counseling, 50 apps (29.1%) for assessment, 12 apps (7.0%) to relieve stress, 24 apps (14.0%) for psychoeducation, and 49 (28.4%) multipurpose apps (ie, a combination of counseling and assessment). Most apps were developed for adults in the general population (166/172, 96.5%), rather than for psychiatric patients. App-based counseling was mostly provided by psychologists, and of the assessed apps, only 40% (70/172) used evidence-based scales to assess mental health problems such as anxiety or depressed mood. Guided meditation was used as the main technique in stress-relieving apps.

**Conclusions:**

Many apps contained useful and evidence-based elements, such as good quality information, validated measurements, and useful meditation methods; however, for mobile apps to contribute significantly to mental health care in China, considerable challenges remain, including the need for more patient-focused apps that can actually take on the role of a health care provider. In addition, efficacy studies are needed.

## Introduction

Mental health problems cause significant distress and have a negative impact on social relationships, school performance, occupational attainment, and physical health [1]. Mental health disorders have been found to affect 12.0% to 47.4% of populations worldwide [2]. In China, the lifetime prevalence of mental health disorders has been found range from 16.6% [3] to 23.6% [4]. Unfortunately, the availability of mental health services in the existing Chinese health care system is currently limited, and many patients cannot obtain appropriate treatment or help for their mental health problems [5,6]. In addition to the lack of available facilities and trained professionals, a low perceived need, poor knowledge of mental health disorders, and mental illness stigma have been identified as potential barriers to seeking help for mental illness [5].

Mobile health (mHealth) technologies could help to partly overcome the above-mentioned obstacles to seeking help, as they could extend the reach of mental health care beyond available health care providers and clinics. The smartphone was one of the most rapidly adopted technological innovations in history [7]; it now provides ubiquitous internet access and the possibility to access, download, and run externally created software apps. As such, smartphone technology provides a unique opportunity to deliver cost-effective and evidence-based mental health care services to large groups of people [8]. Indeed, studies have shown that mental health care apps can play an important role in the assessment, prediction, and monitoring of mental health, as well as psychoeducation delivery, self-management strategies, recovery support, prevention, and promotion. In addition, apps can be used for the training of mental health providers [9-16].

The large number of smartphone users in China—estimated to have reached 748.3 million in 2019 [17]—as well as the large mobile health market in China—which grew by 74.5% between 2016 and 2017 [18]—indicates a large potential for mental health care apps in China; however, very few studies have been done to gain insight into the number and types of available apps and their potential effectiveness in China.

On the one hand, available literature [19-23] seems to suggest limited availability and use of mental health–related apps in China. For instance, a review study [19] on health apps in China found that there were many apps targeting both nonprofessional and professional users, but the study described only 1 app related to mental health. Another review [24] of mHealth technology for the treatment or prevention of mental health disorders in low-and middle-income countries found 6 studies about mHealth technology in China, among which only 1 applied to mental health. On the other hand, an examination of the Chinese iOS and Android app stores shows that, despite a scarcity of research, there is an abundance of publicly available mental health apps. Unfortunately, consumers currently have no access to information about the quality of the available apps beyond the star rating system and user reviews on the app stores. It is unlikely that these indicators of popularity also reflect the quality, effectiveness, or evidence base of an app. Previous studies [20-23] outside of China have found that most mental health apps in commercial marketplaces do not provide evidence-based therapies, do not follow clinical guidelines, and do not respect privacy regulations with respect to the handling of personal information. Consequently, it is a challenge for both patients and clinicians to find useful apps when needed. A recent search of 44 mental health apps (available as of October 2017) in China showed some common features, such as commercial purpose, and services that included counseling, education, and assessment [25]; however, since this review, the market for mental health apps has grown considerably larger (eg, more than 100 available apps were found based on the search term representing mental in the Baidu app store) [26]. In addition, the important characteristics of the apps that are available have remained unclear, such as the target user population, the evidence base for their content, and consideration of data-safety issues. To determine if and how apps can play a role in Chinese mental health care delivery, these aspects should be thoroughly explored, and it is for this reason that this systematic search was undertaken.

The purpose of this study was to (1) characterize the purpose and content of the mental health smartphone apps available for use and most downloaded by the general Chinese public, (2) to evaluate whether app content is evidence-based, and (3) to gain insight into the app costs and into the quality and comprehensiveness of data safety in the apps.

## Methods

### mHealth App Market Search

On December 26, 2018, we conducted a search in both Android (Google LLC) and iOS (Apple Inc) app stores in China. The 3 largest Android app stores in China are Tencent (Tencent Holding Ltd), 360 (Qihoo 360 Technology Co Ltd), and Baidu (Baidu Inc), and these were selected for the study because they constitute more than 50% of Chinese Android market share [27]. In addition, we used the Chinese iOS App store to gather a list of apps for the Chinese iOS market.

We restricted our search to apps that used the Chinese characters for 1 or more of the following keywords in the title or store description of the app: 
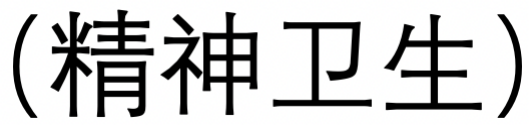
 *(mental health)* OR 
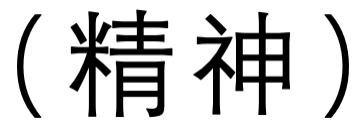
 *(mental)* OR 
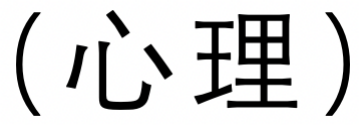
 *(psychology)* OR 
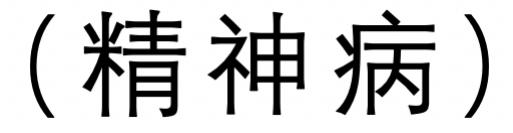
 (*psychiatry)* OR 
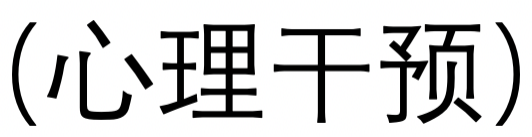
 *(psychological intervention)* OR 
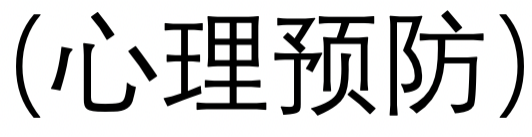
 (*psychological prevention)*. The top 100 apps were collected for each app store.

**Figure 1:**
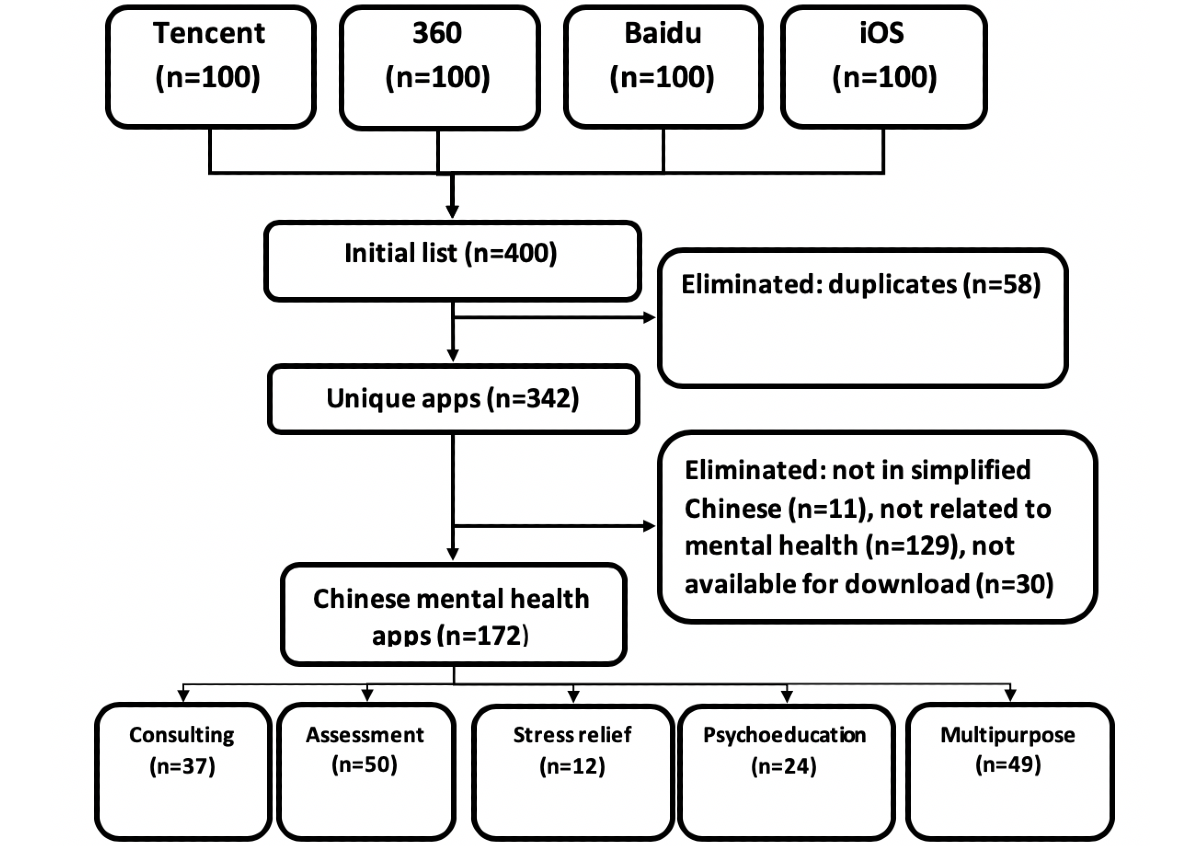
Chinese mental health app inclusion and exclusion flowchart.

Android apps are free to download, and iOS apps are either free or paid. We listed free and paid apps together, mainly because there were few paid apps. The eventual selection of apps for this study (Figure 1) was done according to the following inclusion criteria: The app should (1) be related to mental health, (2) be available for download, (3) use simplified Chinese so that any literate Chinese individual can easily understand the content of the app, and (4) be usable in mainland China.

### App Assessment

The apps that met the inclusion criteria were downloaded onto either a Huawei mate 9 (Android version 8.0.0), a Xiaomi 8 (Android version 8.0.0), or an iPhone 5s (iOS version 7.1) device for a complete assessment. The reason for downloading each app for inspection was that the app description may not be sufficient to allow for a full judgment of the nature and quality of its content. For instance, in a previous study [23], when the authors downloaded the apps, they found that some apps provided patients with poor or potentially harmful advice (such as a recommendation to people with a manic episode to drink alcohol before bedtime to assist with sleeping). Therefore, for each app, we evaluated if the information provided was evidence-based. For apps that were listed in multiple app stores, the consistency of the descriptions across stores was evaluated, and identical duplicates were eliminated. Some apps had a patient version and a clinician version. We assessed these versions separately because they might provide different services. If an app was available for both iOS and Android, the Android version was evaluated.

For each app, the following general information about the app was extracted (Table 1): (1) main purpose, (2) affiliation (ie, commercial, medical center, or university), (3) cost, (4) target user, (5) whether or not information security was emphasized, and (6) whether the therapeutic contents were based on an evidence-based psychological theory, such as cognitive behavior therapy [28], acceptance and commitment therapy [29], interpersonal psychotherapy [30], or problem-solving therapy [31], or if the content was based on advice by professional certified staff including psychologists, psychotherapists, or psychiatrists. Based on the app description, the first author categorized the apps into 1 of 5 categories indicating their main purpose: psychological counseling, assessment, stress relief, psychoeducation, and multipurpose (incorporating components of assessment, education, psychological counseling, and stress relief). Each category of apps was then further investigated by 1 of the coauthors (HY, YW, NW, YZ, and WC; each author was randomly assigned to a category). If anyone found that the initially selected category did not fit an app, the 5 authors discussed until consensus was reached. For each category, specific additional information was extracted (detailed in Table 1). For mental health education apps, core aspects of mental health information (Table 2) were investigated. These aspects have previously been proposed by the National Health Commission of China [32] and are used to guide mental health education work in the whole country.

**Table 1:** The information collected for each of the selected apps.

Information extracted and content		Values
**General information collected for each app**		
	Affiliation	Commercial, university, or medical center.
	Cost	Pay for download, completely free, or partly free.
	Target user	Children or adolescents, general public adults, specific for women, special for patients with mental health problems, or specific for professional staff.
	Data safety information	Whether an app report relevant information security measures or not? (yes or no).
	Professional background	Whether the contents of the app were based on a psychological theory or were based on advice by professional certified staff including psychologists, psychotherapists or psychiatrists? (yes or no).
**Specific information for psychological counseling apps**		
	Counseling mode	Online (text typing or audio) or offline (eg, an app can help to make an appointment).
	Counseling provider	Professional (including psychologists, psychotherapists, or psychiatrists) or trained lay health supporter.
	Target users	Individuals with diagnoses of a mental health disorder or general public.
**Specific information for assessment apps**		
	Assessment aspect	Personality, symptoms, intelligence, cognitive functions, or assessment just for entertainment (eg, constellations, fortune, or other).
	Evidence	Are evidence-based measurements or scales used? (yes or no)
**Specific information for stress relief apps**		
	Methods	Music, mindfulness, relaxation therapy, etc.
	Evidence	Is there a reference in the app to work that shows the therapy is effective?
**Specific information for education apps**		
	Apps for training mental health providers	Books, lectures, audio recordings, or other method.
	Apps for mental health knowledge to public	According to 8 core information aspects (see Table 2).

**Table 2:** Eight aspects of mental health information.

Items	Content
1	Mental health is an integral part of health. No mental illness does not necessarily mean mental health. Everyone needs not only physical health, but mental health as well.
2	Mental health and mental illness, such as physical health and physical illness, are determined by multiple interacting biological, psychological, and social factors.
3	Given that anyone could encounter mental health problems in their life, it is necessary to pay attention to and maintain mental health.
4	Schizophrenia, depression, behavior disorders in children and adolescents, and senile dementia are China's current priorities for mental illness prevention and treatment.
5	A person suffering from psychological or behavior problems or mental illness should seek help from a medical institution as soon as possible to receive advice and formal diagnosis and treatment.
6	Mental illness can be prevented and treated.
7	Care for and do not discriminate against mentally ill patients, and help them return to their families, communities, and society.
8	Mental health is related to social harmony and development. Promotion of mental health and prevention and treatment of mental illness is the responsibility of society as a whole.

## Results

### General

Of the 400 apps that were screened, 172 unique apps met the inclusion criteria (Figure 1). Of these, 105 (61.0%) were available in the Android app stores, 28 (16.3%) were available in the iOS app store, and 39 (37.1%) were available in both the iOS and Android app stores. Based on the descriptions of the main purpose of the apps, 21.5% (37/172) were for psychological counseling, 29.1% (50/172) were for assessment, 7.0% (12/172) were for stress relief, 14.0% (24/172) were for psychoeducation, and 28.4% (49/172) were multipurpose apps. The number of downloads per app ranged from 16 to 107 million in the Android store. Download numbers could not be obtained in the iOS app store. Detailed information about the different groups of apps is shown in Table 3.

### Counseling Apps

There were 37 counseling apps, and they were all developed by commercial companies. Of the counseling apps, 7 (19%) apps were free to use, 29 (78%) apps could be downloaded for free but charged counseling fees to consumers wishing to use the app, and 1 (3%) app charged a download fee. In terms of the target population, 8 (22%) apps were aimed at adults and adolescents, 1 (3%) was aimed specifically at children and adolescents, and 27 (76%) were only aimed at adults. Of the 27 apps for adults, 1 (3%) app was only aimed at women and 3 (8%) apps were aimed specifically at professionals. Of the 37 counseling apps, 20 (54%) apps made explicit claims about information security measures. All of the counseling apps provided some kind of online consultation, which meant that staff was available online to give users feedback and consultation. Counseling by text-based messages was used in 34 (92%) apps. Users could voice-chat with staff in 26 (70%) apps. Of the 37 counseling apps, 20 (54%) provided services to make an appointment with a counselor and 4 (11%) provided services to register appointments at hospitals; 36 (97%) had professionals acting as consultation providers (eg, psychologists, psychotherapists, or psychiatrists), and only 1 (3%) app provided consultation by a trained lay health supporter.

### Assessment Apps

Of the 50 assessment apps, 30 (60%) apps provided information about the affiliations of their developers and all were developed by companies; 44 (88%) were free to use, and the other 6 (12%) were free to download but charged a fee for some of the assessments that were offered. All apps were aimed at adults, and 3 (6%) apps were specifically for women. Only 5 (10%) apps made a claim about information security. Evidence-based scales were used in 17 (34%) apps (some had more than 1 type—12 assessed personality, 8 assessed emotional symptoms, and 1 assessed cognitive ability). The most popular validated questionnaires were the Eysenck personality questionnaire [33,34] used in 8 apps and the Sixteen Personality Factor Questionnaire [35,36] used in 4 apps. The Self-Rating Anxiety Scale [37,38] and Self-Rating Depression Scale [39,40] were used to assess anxious and depressive symptoms in 10 apps. The other 33 (66%) apps were mainly aimed at providing some form of entertainment; they assessed fortunes, mental age, marriage and love, and occupational abilities using assessment items of unknown source.

### Stress Relief Apps

All 12 stress relief apps were developed by companies; 3 (25%) apps were free, and 9 (75%) apps were free to download but charged a fee for some of the content. Of the 12 stress relief apps, only 1 (8%) app made a claim about information security. Of the 12 stress relief apps, 9 (75%) provided evidence showing that meditation is an effective method to relieve stress. Audio was the main medium used in stress relief apps; of the 12 stress relief apps, 1 (8%) app played audio recordings of methods to maintain mental stability from the *Huangdi Neijing* (an ancient Chinese medical text that has been treated as the fundamental doctrinal source for Chinese medicine for more than two millennia), 1 (8%) provided audio of mood control courses, 2 (17%) provided nature sound recordings, and 7 (58%) apps provided audio recordings of guided meditations. Of the 12 stress relief apps, 2 apps (17%) were specifically aimed at children and adolescents and focused on improving their concentration and memory and on relieving examination anxiety, and only 1 (8%) provided an online community function, allowing users to communicate with each other to relieve stress. Meditation apps used different techniques such as respiration exercises (5/7, 71%), gradual relaxation exercises (7/7, 100%), and biofeedback therapy (1/7, 14%).

### Psychoeducation Apps

All 24 psychoeducation apps were developed by commercial companies. Of the 24 psychoeducation apps, 23 (96%) were for adults of which 4 (17%) were targeted at mental health professionals and provided training courses or e-books about mental health, and the other 19 (79%) were aimed at the general population to provide education and knowledge about psychology and mental health. All of these apps were free to download; however, 3 of the apps for professionals charged a fee for some of the courses in the apps. None of the psychoeducation apps made a claim about data security. Of the apps aimed at the general population, 11% (2/19) were e-books about behavioral psychology and general psychology, 5% (1/19) provided a list of psychological website links, 5% (1/19) provided audio recordings about mental health, 5% (1/19) provided case descriptions showing users the how mental health disorders can manifest, 5% (1/19) provided information about the diagnostic process of mental health disorders, and 74% (14/19) apps included psychological popular science articles, in which common mental health problems in parenting, in the workplace, and in marriage and love were covered, and coping methods were described. We scanned the articles in the 14 apps to check whether they referred to any of the eight aspects of mental health information and found that 10 out of 14 referred at least one aspect, and 9 out of 14 apps covered all of the eight aspects.

**Table 3:** The characteristics of apps in different categories.

Content characteristics			Counseling (n=37), n (%)		Assessment (n=50), n (%)		Stress relief (n=12), n (%)		Psychoeducation (n=24), n (%)		Multipurpose (n=49), n (%)	
**Affiliation**												
	Commercial company			33 (89)		30 (60)		11 (92)		14 (58)		43 (88)
	University			0 (0)		0 (0)		0 (0)		0 (0)		1 (2)
	No information			4 (11)		20 (40)		1 (8)		10 (42)		5 (10)
**Cost**												
	Pay for download			1 (3)		0 (0)		0 (0)		0 (0)		0 (0)
	Fully free			7 (19)		44 (88)		3 (25)		20 (83)		10 (20)
	Partly free			29 (78)		6 (12)		9 (75)		4 (17)		39 (80)
**Target user**												
	Adults and children			8 (22)		0 (0)		2 (17)		0 (0)		7 (14)
	Only children or adolescents			1 (3)		0 (0)		0 (0)		1 (4)		4 (8)
	**Only adults**											
		General adults		24 (65)		47 (94)		10 (83)		19 (79)		37 (76)
		Women		1 (3)		3 (6)		0 (0)		0 (0)		0 (0)
		Patients with mental health problems		0 (0)		0 (0)		0 (0)		0 (0)		0 (0)
		Professional staff		3 (8)		0 (0)		0 (0)		4 (17)		1 (2)
**Data safety information**												
	Yes			20 (54)		5 (10)		1 (8)		0 (0)		20 (41)
	No			17 (46)		45 (90)		11 (92)		24 (100)		29 (59)
**Professional background or evidence-based**												
	Yes			36 (97)		17 (34)		9 (75)		24 (100)		45 (92)
	No			1 (3)		33 (66)		3 (25)		0 (0)		4 (8)
**Counseling mode**												
	Only online			13 (35)		—		—		—		24 (49)
	Only offline			0 (0)		—		—		—		0 (0)
	Both			24 (65)		—		—		—		15 (31)
	None			0 (0)		—		—		—		10 (20)
**Counseling provider**												
	Professionals			36 (97)		—		—		—		37 (76)
	Trained lay health supporter			1 (3)		—		—		—		2 (4)
**Assessment aspect^a^**												
	Personality			—		12 (24)		—		—		21 (43)
	Emotional symptoms			—		10 (20)		—		—		33 (67)
	Intelligence			—		0 (0)		—		—		0 (0)
	Cognitive function			—		1 (2)		—		—		0 (0)
	Entertainment			—		36 (72)		—		—		22 (45)
**Stress relief methods**												
	Music			—		—		2 (17)		—		4 (8)
	Meditation			—		—		7 (58)		—		6 (12)
	Course			—		—		1 (8)		—		1 (2)
	Audio books			—		—		1 (8)		—		1 (2)
	Online community			—		—		1 (8)		—		2 (4)
**Psychoeducation content (eight aspects)^b^**												
	Mental health is an integral part of health			—		—		—		10 (42)		35 (71)
	Multiple interacting factors determinants			—		—		—		9 (38)		26 (53)
	Anyone could encounter a mental health problems in their life			—		—		—		10 (42)		32 (65)
	China's current priority mental illnesses			—		—		—		10 (42)		26 (53)
	Go to the medical institutions for treatment			—		—		—		10 (42)		33 (67)
	Mental illness can be prevented and treated			—		—		—		10 (42)		32 (65)
	Care for and do not discriminate against mentally ill patients			—		—		—		10 (42)		30 (61)
	Promotion of mental health is the joint responsibility of society			—		—		—		10 (42)		30 (61)

^a^These subheading values do not add to n or 100% because it was possible for an app to assess more than one aspect.

^b^These subheading values do not add to n or 100% because it was possible for an app to address more than one item.

### Multipurpose Apps

Of the 49 multipurpose apps, 44 apps (90%) provided affiliation information; 1 (2%) was developed by the Institute of Psychology of Chinese Academy of Sciences, and the other 43 (88%) were developed by companies. Of the 49 apps, 38 (78%) were mainly aimed at adults, 7 (14%) were for individuals of any age, and 4 (8%) were for children and adolescents. Of the 38 apps for adults, there was an app (*Guan Ai ++*) for people in the workplace, an app (*Guan Ai Ji Ceng Gan Bu*) for community-level officials, and an app (*Hao Xin Qing*) that had a version for clients and a version for clinicians. Of the 49 multipurpose app, data safety claims were found for 20 (41%) apps. All the multipurpose apps could be downloaded for free, but users were charged an additional fee for some content in 39 apps (80%). Most multipurpose apps (45/49, 92%) involved professional staff, evidence-based therapies, or references to evidence-based materials.

Consulting, assessment, psychoeducation, and stress relief were combined in 5 (10%) multipurpose apps; consulting, assessment, and stress relief were combined in 2 apps (4%); consulting, assessment, and psychoeducation were combined in 19 (39%) apps; none combined counseling and assessment; counseling and psychoeducation were combined in 5 (10%), assessment and stress relief were combined in 2 (4%), assessment and stress relief were combined in 2 (4%), and stress relief and psychoeducation were combined in 3 (6%).

Of the 49 multipurpose apps, 39 apps (80%) provided some kind of online counseling; with the exception of 2 apps, counseling services were provided by mental health professionals (31 apps involved a psychologist or psychological therapist; 6 apps involved a psychiatrist). In several apps, users could choose which professional to have counseling with, after looking through the profiles of listed professionals. Depending on the app, users could contact the professional by telephone, online video or voice chat, text-based messages, or they could make an appointment with the professional; users had to pay a fee, depending on the price of the listed professional. Two apps offered online consults with a professional but also included a system to make an appointment with a professional at a hospital. One app (*Hao Xin Qin doctor version*) aimed to help professionals to manage their clients online.

Of the 49 multipurpose apps, 41 apps (84%) had some sort of assessment functionality; 80% (33/41) used evidence-based self-report questionnaires to evaluate personality (2 used the Eysenck personality questionnaire, and 1 used the Sixteen Personality Factor Questionnaire), anxiety (14 apps used the Self-Rating Anxiety Scale), and depression (26 apps use the Self-Rating Depression Scale, 1 app uses the Hamilton depression rating scale [41], and 1 used the Symptom Checklist-90 [42]), or social phobia (items from the Liebowitz social anxiety scale)[43]. Some entertainment assessments such as fortune, love, and marriage were also included in 23 apps. None of the apps aimed to assess intelligence or cognitive abilities.

Of the 49 multipurpose apps, 14 (29%) of the multipurpose apps had stress relieving sections and provided audio recordings of relaxing music or meditation guidance, and 36 apps provided either psychoeducation articles or audio recordings related to the eight key aspects of mental health knowledge.

There were 4 (8%) multipurpose apps specifically for children and adolescents; 1 app (*jie you nuan xin mao*) focused on assessing everyday mood, providing counseling services, and had the option for discussion with fellow users; 1 app (*xin li mei*) was developed specifically for students in a specific region’s primary and secondary schools, providing multiple services including assessment, stress relief, counseling, and making appointments with a teacher-counselor; 1 app (*Q xin li*) provided counseling, questions and answers, microlectures, and psychoeducational articles for users; and 1 app (*Gao kao jian ya bao*) was developed to help students to reduce stress induced by college entrance examinations by providing counseling, stress-reducing music, and assessment.

## Discussion

### Principal Findings

To our knowledge, this is the first study to search and evaluate the contents of the full range of available and most widely used mental health smartphone apps in China. Evaluation of each of the apps showed different primary aims, with most focusing on assessment, counseling, and a combination of multiple purposes (ie, assessment, counseling, stress relief, and psychoeducation). Most apps were developed for profit and focused on the adult population. Only 6 apps were specifically aimed at children or adolescents. A majority of the apps provided counseling or assessment, often enlisting online psychological counseling services from professionals such as psychologists, psychotherapists, or psychiatrists; however, even though psychometrically valid assessments of personality, anxiety, and depression were included in some assessment apps, assessments in most apps were more for entertainment than for assessment of psychiatric symptoms. Audio-recordings to guide or accompany meditation were the main materials provided by the apps that focused on stress reduction. Most psychoeducation apps provided information that aligned with national guidelines on mental health information.

Evaluation of the target populations for the reviewed apps provided clear insight into the perceived market opportunities by app developers. This study showed that developers of mental health apps prefer to target the general population rather than patients with mental health disorders: No reviewed app focused specifically on patients with mental health disorders. In addition, although there was a small number of mental health apps for specific adult populations (eg, women, workplace, staff) or for mental health professionals, most apps were aimed at a broad audience. This is understandable given that most developers will want to acquire as many users for their app as possible, but this does leave room for apps that focus on assisting patients with mental health disorders. Interestingly, only a few apps were developed for children and adolescents. This does not reflect the current situation, where the prevalence of mental health disorders in children and adolescents in China lies close to the high worldwide prevalence of 20% [44]. In fact, the mental health of children and adolescents is a point of attention for both policy maker and researchers [45]. The fact that app developers have largely ignored this population could be due to various reasons. For instance, app developers may be put off by worries in the public about negative effects induced by mobile phone use [46]. In addition, children and adolescents may be a less attractive market because many parents and schools impose time restrictions on smartphone use by children and adolescents [47].

Few apps provided special interventions for patients with mental health disorders. This may partly be motivated by market considerations (ie, smaller target user population), but could also reflect the current lack of consensus about evidence-based guidelines for mHealth therapy for mental health patients. There is a large body of international literature about the use of mHealth interventions for people with mental health disorders [11,48-50] and similar work has been carried out in China [51]; however, the development of smartphone-based therapy is still in quite an early phase, and it may indeed be too early to offer it outside an experimental or academic mental health care setting. At this point, there is still a long way to go, both in terms of app development and efficacy evaluation, before evidence-based mHealth therapy can be routinely offered to patients in China.

With the exception of one, all apps could be downloaded for free; however, the actual cost of the services offered depended strongly on what was provided in the apps. In this study, more than 70% of the counseling apps, stress relief apps, and multipurpose apps charged a fee for some services, whereas a large part of the assessment apps and psychoeducation apps were free to use. This might be because counseling apps, stress relief apps, and multipurpose apps involved more user-specific services, offered either by paid professionals or as part of evidence-based stress relief methods that require a larger initial investment from the developer than, for instance, the inclusion of articles about mental health or the use of questionnaires that have developed by others. The cost of certain services in apps might present an obstacle for users, but this issue was not within the scope of this study.

Assessment was the most popular function across all apps; there were 49 assessment apps and 41 multipurpose apps that had an assessment section; however, most assessment apps (or sections) focused on assessment as a form of entertainment rather than on the assessment of real psychological problems or characteristics. Most multipurpose apps that also included counseling and stress relief sections, used assessments with psychological scales, whereas entertainment assessments were included in most single purpose assessment apps. Assessments of personality, anxiety, and depression were most common in apps that used psychological assessment scales, which makes sense given the high prevalence of anxiety and depression in China [3,4] and aligns with previous observations that people with anxiety and depression often do not seek help from professional institutes or physicians [52]. It could be that this group is relatively more likely to use a freely available mobile app for self-assessment.

Psychological counseling was the second most popular service and was provided in all 37 counseling apps and in 39 multipurpose apps. Psychologists were the main providers of counseling. Only 16 apps involved psychiatrists as providers of counseling. This indicates that psychologists in China play an important role in delivery of mental health care to the general population. This could be due to their larger number, which makes it easier for users to find a psychologist for mental health services. Indeed, in 2017, it was estimated that approximately 40,000 people in China held a national second- or third-level certificate in psychological counseling and provided psychological counseling on either a full- or part-time basis [53]. In contrast, the number of certified psychiatrists in China (in 2015) was 27,733. Another explanation for the relatively large number of psychologists involved in the reviewed apps could be that regulation in China leads psychologists and psychiatrists working in different settings. Psychologists work in a broad range of settings, whereas all psychiatrists in China need to be registered to a mental health hospital or psychiatric ward in a general hospital that provides inpatient service [5]. According to the Mental Health Law of the People’s Republic of China [54] psychologists should only perform counseling and are not authorized to perform psychotherapy, nor engage in the diagnosis or treatment of mental health disorders. If psychologists detect that a person receiving counseling may suffer from mental health disorders, he or she should recommend that the person seek services at a medical institute. As such, online counseling is a good way for psychologists to perform initial counseling in population-dwelling persons; however, it is currently hard to estimate the effectiveness of online psychological counseling by psychologists in China because of serious problems with the level of professionalism of psychological counseling (low performance levels, a lack of norms, and uneven levels of training and expertise across consulting staff) [55]. Unfortunately, we did not have any access to information about the kinds of problems users seek treatment for via the apps, nor did we have insight into the number of users that should be and the number that were, in fact, referred to medical care by consulting psychologists.

Another important finding was that most mental health apps simply served as a means to connect help seekers to health care providers, but did not use digital technologies (such as interactive computer-guided treatment algorithms) to deliver mHealth interventions that could (partly) replace a health care provider. This is not a problem per se, but it means that existing apps are unlikely to solve the current problem of insufficient mental health care providers for the demand in China. To achieve that, apps that can (partially) provide services that would otherwise be provided by health care professionals should be developed. More and more apps are being developed that offer a completely digital interactive therapy environment. For instance, cognitive behavior therapy–based mHealth apps have been developed for a large range of mental health problems, and the efficacy of such interventions has been confirmed in several studies [56-60]. To the best of our knowledge, such apps have not yet been developed for the Chinese population, nor have they been tested.

This study showed that meditation was the main intervention offered by apps that aim to help users relieve stress. Previous studies [61,62] have, indeed, shown that meditation supported by online tools can have a significant beneficial effect on depression, anxiety, and well-being and a large effect on stress. This seemed to be at least partially supported by the evidence-based nature of these apps; however, no research exists on the efficacy of mobile app–assisted meditation, specifically in China.

Most psychoeducation apps covered one or more of the eight core aspects of mental health information that have been proposed as part of national policy. As a result, the apps were usually quite complete and accurate in their information coverage. The fact that some psychoeducation apps were downloaded more than 100,000 times (eg, the app *geilixinli* had been downloaded 780,000 times, and the app *xinlizixunyidianling* had been downloaded 580,000 times), indicates that mHealth psychoeducation can play an important role in disseminating mental health knowledge, which in turn could help reduce levels of depression and psychological distress [63,64].

### Limitations

This study has some limitations. First, we could not obtain the number of downloads for all apps because the iOS app store does not provide this information. In addition, even for the apps for which the download numbers were known, we could not be sure about the number of persons who actually used the app. Second, we based the evaluation of the apps on quite a superficial screening the app appearance and the information delivered by the developers. We did not check and cannot be sure that all services (eg, contact with certified professional) are provided as claimed and that all apps function correctly. Third, we evaluated if apps made any statements or claims about data safety, but could not evaluate if they, in fact, handled data securely. Fourth, we chose a set of search terms to make sure that we included many relevant apps; however, it is still possible that we missed some potentially relevant apps. Fifth, we did not carry out a formal evaluation of each app’s quality as this was outside the scope of this study. This study looked at the landscape of services and functionalities that are provided by the apps and evaluated their potential to contribute to mental health care development; however, app quality is also an important factor in determining an app’s usefulness. An interesting possibility for future studies could be to use a standardized measure to assess and compare the quality of individual apps (eg, the mobile app rating scale [65]); indeed, the mobile app rating scale has been used to evaluate the quality of mobile apps in other health-related fields in China [66]. Proper translation and validation of scales such as the mobile app rating scale in Chinese would be extremely useful for the systematic evaluation of mental health care apps. Sixth, all available apps were evaluated as of December 2018. Apps that have been newly added since then have not been included in this study; however, a new search (May 23, 2020) for the top 100 apps in the three Android app stores revealed that only 9 apps had been added since the initial search, making it unlikely that the current results and conclusions would have been significantly affected by their inclusion.

### Conclusions

This study uncovered a large number of mental health apps available in China. The results show that there is a large variety in the aims and evidence-base among the apps. A considerable number of apps contained useful and evidence-based elements, such as good quality information about mental health, validated questionnaires, useful meditation methods, and access to counselors, which makes it likely that they can contribute positively to mental health care in China; however, for mental health care apps to substantially contribute, considerable challenges remain, including the need for more patient-focused and child- and adolescent-focused apps, the development of apps that can take over some of the roles of a health care provider, and efficacy studies.

## Abbreviations

mHealth: mobile health
